# Team Efficiency in Organizations: A Group Perspective on Initiative

**DOI:** 10.3390/ijerph17061926

**Published:** 2020-03-16

**Authors:** Ana Lisbona, Abel Las-Hayas, Francisco J. Palací, Miguel Bernabé, Francisco J. Morales, Alexander Haslam

**Affiliations:** 1 Departamento de Psicología Social y de las Organizaciones, Universidad Nacional de Educación a Distancia, Juan del Rosal 10, 28040 Madrid, Spainfpalaci@psi.uned.es (F.J.P.); mbernabe@psi.uned.es (M.B.); jmorales@psi.uned.es (F.J.M.); 2 University of Queensland, School of Psychology, St Lucia QLD 4072, Australia; a.haslam@uq.edu.au

**Keywords:** personal initiative, climate for initiative, social identification, team work engagement, innovation, productivity, teams

## Abstract

Organizational research has shown that personal initiative is related to both climate for initiative and work engagement. Yet little is known about what happens to this relationship once the focus shifts to the team level. When organizational and team goals are involved this renders the relationship more complex, and team identification and organizational identification are likely to be key to understanding it. In this paper we develop a model to deal with these complexities. This predicts (a) that team identification will impact on team initiative through team work engagement while (b) organizational identification will impact on team initiative through climate for initiative. It is also expected that team initiative will, in turn, impact on team productivity, and on radical innovation as evaluated by the team leader. This model was tested in a field study with 327 participants of 76 workteams belonging to 50 organizations. Results of SEM and regression analysis supported our main hypotheses. Findings showed that initiative is related to performance and also underline the importance of initiative at a team level. At the same time they suggest that to develop teams with high levels of initiative it is important to promote both organizational and team identification.

## 1. Introduction

In the current changing competitive work environment, where markets, society and customers have higher expectations than in the past, workplaces demand a high level of employee flexibility, rapid innovation, and efficient implementation of new products, new ideas, new services, and new forms of work [1]. Accordingly, innovation and efficiency are generalized requirements in the modern workplace. Organizations that acknowledge this point therefore embark on the hunt for better managerial practices and, likewise, many scholars have become increasingly interested in the question of what drives team innovation and performance [2,3,4,5,6]. 

Organizations require engaged employees who are self-efficacious, proactive and show a high degree of active performance at work [7]. Being proactive involves taking control to make things happen rather than waiting for things to happen or hoping that they happen [8,9]. Making things happen involves aspiring and striving to bring about change and with a view to creating a better future [10,11].

This fact has spurred an interest in work engagement [12] and the development of new concepts such as personal initiative [13]. Yet as Williams et al. [3] note, most research and theory in this area has focused on individual employees, and the concept of proactivity, or active performance has also largely been considered at the individual level. Indeed, most discussions of proactive behavior focus on self-initiated, future-focused action in which individuals aim to change some aspect of the external situation (e.g., work methods) or some aspect of themselves [8] (e.g., their preparedness for particular challenges).

To achieve or improve active performance, it is clearly the case that individuals matter. However, in many organizational contexts teamwork is equally—and often more—important. Furthermore, because few jobs involve working entirely on one’s own, a group perspective is often demanded. In addition, working in teams can have a range of advantages. In particular, it may (a) offer opportunities for job enrichment, (b) decrease the workload of supervisors, and (c) increase performance on tasks that are too complex for individuals [2]. At its best, teamwork thus offers a way of synthesizing individuals’ knowledge, skills and abilities in order to achieve exceptional creativity, innovation and productivity [14]. As Fay et al. [4] observe “the optimistic rhetoric of teamwork argues that the more widespread teamwork is in an organization, the higher the level of organizational innovation”.

Nevertheless, few studies have focused on what drives this active performance behavior at the team level [3]. While there is a large body of research on innovation in teams which focuses on team characteristics, processes, members, environment and work organizations as well as on how all thee factors affect teams, few studies have looked at the dynamics of teamwork in organizations to see how these affect an organization’s innovativeness [4]. To get a full understanding of this issue, we argue that team and organizational levels of initiative should be researched together. 

Within the context of proactivity or active performance, Williams et al. [3] proposed a team-level concept: *team proactive performance*. Along the same lines, several authors have suggested that engagement is a powerful antecedent of performance in organizations, implying that it should not be regarded exclusively as an individual-level construct, but also as a team-level phenomenon [14,15,16,17]. But while there are high-performance teams—and organizations—there are also teams that perform worse than would be the case if the individuals in them were to work alone. So what produces the spark that induces team and organizational members perform better? One body of research that has the capacity to generate answers to this question is informed by theorizing in the tradition of the *social identity approach* [14,18].

### 1.1. Team Initiative, Team Work Engagement and Team Identification

Personal initiative is a form of proactive behavior [10]. Despite the fact that research and practice have pointed to the key role played by teamwork in the achievement of organizational efficiency and competitiveness [19,20], to date proactive behavior scholarship has largely been focused on the individual level. Nevertheless, there are some exceptions—notably in work on team proactive performance [3], team initiative [21,22,23] and organizational climate for initiative [24].

Team initiative is a collective construct that, like personal initiative [25], is defined as a behavioral syndrome that results in a workgroup taking an active and self-initiated approach to the pursuit of work goals and tasks and persisting in overcoming barriers and setbacks [23]. As a collective phenomenon, team initiative can be understood as a shared team attribute that originates in the experiences, attitudes, perceptions, values, cognitions and behaviors that team members have in common. 

The concepts of work engagement and personal initiative have been studied in separate literatures that have hitherto had minimal impact on each other. However, at least three different studies have identified a positive relationship between work engagement and personal initiative [26,27,28]. When a team perspective is adopted, much as in the case of team initiative, team work engagement is defined as a positive, fulfilling, work-related and shared psychological state characterized by collective vigor, dedication and absorption which emerges from the interaction and shared experiences of the members of a workteam [17].

As suggested by Strauss, Griffin and Parker [29], research has focused extensively on self-efficacy as a driver of proactive behavior (e.g., [30,31,32,33]). In their model of proactive motivation Parker et al. [33] refer to this as a “can do” pathway. Similarly, self-efficacy is related to work engagement [34,35]. These lines of research suggest that proactive behavior and work engagement are driven by people’s belief that they can perform a particular task (or set of tasks). Nevertheless, we still need to explain *why* people are proactive. Less research has explored this “reason to” pathway [8] but as we noted above, Richardson and West [14] suggest that social identity theorizing might be relevant here as it explains why individuals are motivated to engage with groups and organizations. Related to this point, Parker et al. [8] refer to the role of identity in the motivation of proactive behavior, arguing that, over and above finding a proactive behavior enjoyable, individuals engage in proactive action because this is fundamental to their identity (see also 18). Along these lines, Ellemers, De Gilder, and Haslam [2] also argue that social identity theorising provides a platform for understanding behavioral motivation in contemporary work settings primarily because it provides a framework for understanding how goals become integrated into a person’s sense fo self (see also [36,37,38,39,40]).

The potential relevance of social identity theory [40] for organizational scholarship was first discussed by Ashforth and Mael in 1989 [41]. Since then, there have been a range of applications of the theory to organizational phenomena. As a result, the theory has emerged as an important framework for understanding the psychology of individuals in organizations [42,43]. Indeed, it has been argued that it is social identity that makes organizational life possible [44].

Social identification is the process by which group memberships are internalized into a person’s sense of self (so that their sense of self is defined in terms of “we” and “us”, rather than just “I” and “me”). Social identity thus refers to a person’s readiness to define himself or herself in *depersonalized* terms as a member of a particular group. When a particular social identity is salient this leads people to see characteristic features of that group membership to be self-defining and to adopt distinctive group norms as a guide for their own behavior [2]. In this way, across a range of social and organizational contexts the groups to which we belong shape our understanding both of who we are and how we should behave [45,46]. 

Richardson and West [14] note that when a person defines themselves in terms of social identity and group norms prescribe high levels of engagement on a team task or a high level of team initiative, then this is likely to stimulate greater behavioral effort on behalf of the team [36]. Moreover, when there is a high level of team identification, a degree of social interdependence or cohesion is created, reflecting the psychological bond and attraction of team members to one another, and to the team as a whole. Here, social identification will also lead team members to develop an inherent concern for the needs of the group and to be more willing to make the psychological investment (e.g., in the form of loyalty [47] and dedication to their team) that is required to build team engagement and team initiative. In line with this idea, research has found that employees’ vigor is predicted by workgroup cohesion [48].

Related to this analysis, Tyler and Blader’s group engagement model [49,50] holds that social identity is necessary to understand the psychological basis of people’s engagement with groups, organizations, and societies. The insights derived from this model have been integrated into a large body of organizational research and theorizing (e.g., [2,51,52,53,54,55,56]). In other work, team identification is also understood to be related to proactive behavior, with research showing that social identity is a stimulus for extra-role behavior [49]. Similarly, workgroup social identity has been found to explain the relationship between extra-role behavior and employees’ procedural justice judgments as well as their economic outcome judgments [46]. Moreover, recent research [57] has found that leaders’ *identity entrepreneurship*—the degree to which leaders work to increase members’ sense of shared social identity—leads to enhanced work engagement on the part of group members which in turn strengthens the positive impact of work engagement on group performance. 

On the basis of these inter-related strands of previous research, we therefore derive the following hypotheses:

**H1a:**  *Team identification will be positively related to team work engagement.*

**H1b:**  *Team work engagement will be positively related to team initiative.*

**H1c:**  *Team identification will have an indirect positive relationship with team initiative via team engagement.*

### 1.2. Climate for Initiative and Organizational Identification

The theoretical model of antecedents and consequences of personal initiative highlights environmental support as a personal initiative antecedent and research has also focused in climate for initiative as an organizational support antecedent. Climate for initiative is an organizational-level construct which refers to formal and informal organizational practices and procedures that guide and support a proactive, self-initiated, and persistent approach toward work [21]. Active coping is another antecedent of personal initiative and organizational identification which can function as a valuable resource when it comes to coping with stressors [58].

Extra-role behavior or OCB are concepts related to active performance, proactive behavior or personal initiative. In particular, several studies have observed links between organizational identification and OCB or extra-role behavior (e.g., [59,60]). For example, van Dick, van Knippenberg, Kerschreiter, Hertel and Wieseke [61] found work group and organizational identification to have an interactive impact on extra-role behavior. This line of research therefore leads us to the following hypotheses:

**H2a:**  *Organizational identification will be positively related to climate for initiative.*

**H2b:**  *Climate for initiative will be positively related to team initiative.*

**H2c:**  *The effect of organizational identification on team initiative will be mediated by climate for initiative.*

### 1.3. Team Initiative Outcomes: Innovation and Productivity 

After reflecting on the antecedents of team initiative, we turn next to its consequences. According to the literature, individual and organizational performance are the principal outcomes of personal initiative [13]. More specifically, research has observed a positive relationship between (a) personal initiative and innovation [62,63,64,65] as well as between (b) team initiative and productivity and (c) climate for initiative and innovation [23].

Personal initiative is a behavioral syndrome that involves being pro-active and developing goals that go beyond what is formally required in one’s job. In particular, this involves thinking about long-range problems and developing long-term goals and plans [13]. In this, personal initiative goes beyond creativity because when a person shows personal initiative it is necessary not only have a great new idea but to *implement* it. The result of this should be innovation. In line with this logic, Fischer et al. [64]. distinguish between two types of innovation: incremental and radical. Radical innovation is disruptive because the process of introducing something new makes existing knowledge and practices obsolete [64,66]. Radical innovation also gives rise to fundamental changes in the activities of an organization or industry with respect to current practices: “it poses new questions, develops new technical and commercial skills, and new ways of resolving problems.” [67] (p. 336). 

In line with this logic, we consider radical innovation to be an organizational performance outcome. This accords with work by Fischer et al [64] which found that climate for initiative was related to radical innovation, but not to incremental innovation. Likewise, the definition of personal initiative leads us to expect that its proactive dimension will lead to radical rather than merely incremental innovation [23].

Consistent with this reasoning, William et al. [3] note that team proactive behaviors or self-initiated strategies to manage performance barriers or proactivity in problem solving are positively related (a) to team-level customer service and productivity [68], (b) to more effective teams [69] and (c) to team learning and team performance in short-term student project teams [70]. This leads us to the following hypotheses:

**H3a:**  *Team initiative will be positively related to productivity.*

**H3b:**  *Team initiative will be positively related to innovation.*

### 1.4. Modelling the Relationship between Antecedents and Consequences of Team Initiative 

In organizational contexts, groups provide employees with a sense of self by furnishing them with a sense of social identity defined at a particular level of abstraction (e.g., at a subgroup level as a member of a workteam or at a superordinate level as a member of an organization). Abrams et al. [71] argue that in these contexts people often project the attributes of lower-level (e.g., subgroup) identities onto the superordinate identity. This indeed is a key way in which an organizational norm becomes accepted as an ingroup norm. At the same time, this process can also satisfy motivations for (a) self-enhancement and (b) uncertainty reduction [72]. Nevertheless, there are some contexts in which workteam identity will be more salient than organizational identity (e.g.., when working on a particular team project) and others where organizational identity will be more salient than workteam identity (e.g., when one’s organization is merging with another [73,74,75]). 

These observations speak to the fact that the dynamics of identity salience are complex [73,74,75]. As evidence of further complexity, researchers have observed that social identities can sometimes give rise to counterproductive behavior in workteams (e.g., in the form of soldiering [76]), where a group sets norms for underperformance [77]. At the same time, the desire to establish a distinctive social identity may direct group members’ efforts toward activities that set them apart from other groups in ways that have negative consequences for the organization as a whole. For example, as Ellemers, de Gilder and Haslam [2] point out, this motivation for distinctiveness will tend to compromise group productivity if it is associated with systematic underperformance or excessive absence. Along similar lines, Van Knippenberg and Van Schie [78] point out that strong identification with a workgroup will not always be beneficial for the organization as a whole. Indeed, because identified and committed group members internalize their group’s norms they are likely to follow these norms even when they are in conflict with those of the larger organization. González-Romá and Hernández also note that increased group identification can sometimes increase the amount of effort directed towards the achievement of individual goals when distinctive group norms prescribe individualistic behavior [79]. To address this, they recommend setting distinct goals for subgroups so as to foster a sense of identification with the group and also increase members’ efforts to achieve these group goals [77]. 

Meta-analytical evidence presented by Rikketa and Van Dick [80] has explored the determinants of these different foci of identification across a broad range of organizational contexts. As other social identity theorists (e.g., [57,73,74,75,78]) have proposed, these researchers found that workgroup attachment was not always more important than organizational attachment in accounting for work-related attitudes and behaviors. Moreover, team-related variables, such as team climate perceptions, satisfaction with co-workers or supervisors and altruistic behaviors, were more closely related to workteam attachment than to organizational attachment. On this basis, Rikketa and Van Dick [80] argue that the focus of attachment (i.e., the locus of social identification), should be central to attempts to explain differences in work-related attitudes and behaviors. At the same time, we suggest that team and organizational constructs are not independent but rather need to be understood as interrelated in ways suggested by the subjective group dynamics model [71,81,82]. This model follows social identity theory in arguing that group members strive to ensure that ingroups (e.g., workteams) are superior to outgroups, and follows self-categorization theory in suggesting that the categorization process is largely driven by a search for meaning [71]. And here again the motivation to maximize and sustain positive intergroup distinctiveness is likely to be a key driver of group norms and the behavior that flows from them [71]. In order to do justice to these complexities, our goal in this study is to explore the impact of *both* team and organizational identification on innovation, in light of previous evidence that negative consequences can flow from an exclusive focus either on innovation or on team performance with disregard for organizational outcomes. 

**Figure 1:**
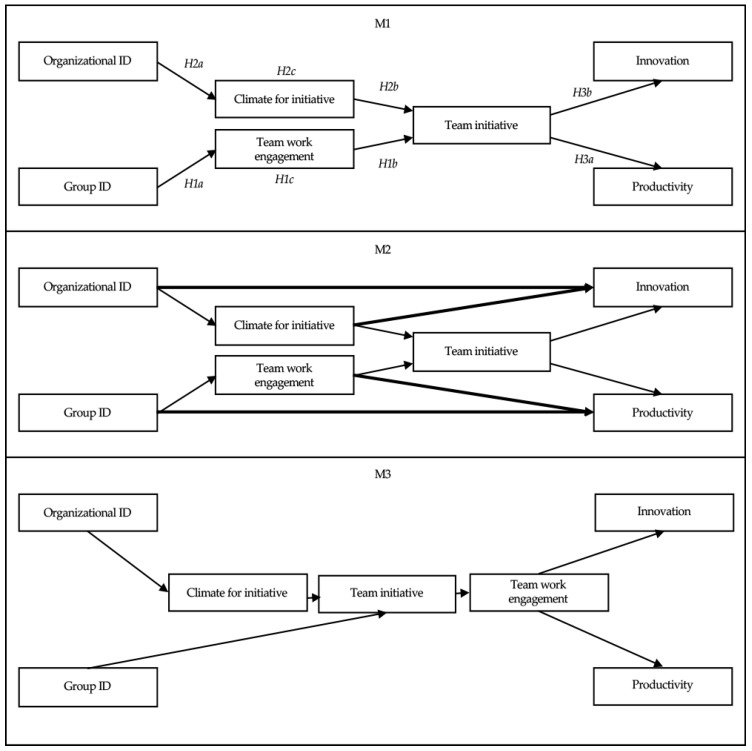
Theoretical model.

In summary, then, we propose a model of team innovation that integrates all the above hypotheses. The aim of the present research was to provide a preliminary test of this model. More specifically, we sought to examine the relationship between both organizational identification and team identification and both innovation and productivity, as well as the role of climate for initiative, team work engagement and team initiative in mediating between them. These hypotheses are represented schematically in Model 1 (M1) in Figure 1.

In this, the objectives of the study were to understand how team initiative is related to productivity and innovation and how team initiative is influenced by different aspects of organizational complexity. This complexity arises at two levels of abstraction. First, at the organizational level where organizational identification and organizational climate are hypothesized to impact on team initiative; second, at the group level where team initiative is hypothesized to depend on group identification and team work engagement. We also seek to establish whether the team initiative that results from these two levels of input is related to two key outcomes: (a) productivity and (b) radical innovation. 

## 2. Materials and Methods

### 2.1. Samples and Procedure

The sample consisted of 327 employees (52% men and 48% women) in 76 workteams belonging to 50 organizations, 251 team members and 76 leaders. Demographic details are provided in Table 1. 

Organizations were invited to take part in the study and participation was voluntary. Participants were informed about the objectives of the research, its voluntary nature, and the anonymous and confidential use of the data. Participants completed questionnaires after providing informed consent.

The aims and hypothesis of the study were not disclosed to the contact person in each organization or to participants. The researcher simply said that the aim of the research was examine how work teams functioned. Participants were provided with a definition of work team and the inclusion criteria for teams was also explained. For this purpose, we drew on Kozlowski and Bell’s [19] definition of work team as a group of two or more individuals who (a) socially interact (face-to-face or, increasingly, virtually); (b) possess one or more common goals; (c) are brought together to perform organizationally relevant tasks; (d) are interdependent with respect to workflow, goals, and outcomes; (e) have different roles and responsibilities; and (f) are embedded in an encompassing organizational system, with boundaries and linkages to the broader system context and task environment. Five additional inclusion criteria were established for teams: (a) being active and having a minimum activity of 6 months, (b) being composed of a minimum of 3 members (excluding the leader), (c) obtaining completed questionnaire from at least 2 members of every team, (d) having a single team leader and (e) having a maximum of 5 different teams per organization.

### 2.2. Operationalization of Variables

To reduce Common Method Bias [83], we obtained measures of the predictor and criterion variables from different sources using two questionnaires: one for team members to assess predictor variables and another for leaders to assess criteria variables.

#### 2.2.1. Measures from Team Members

Organizational and group identity was assessed by seven items [59], e.g., ‘I am proud to belong to ... (... my Organization.)’ / ‘I am proud to belong to ... (... my Group.)’; α = 0.94 and α = 0.96). Participants responded to these on 6-point Likert scales ranging from 1 (completely disagree) to 5 (completely agree).

Climate for initiative was assessed by seven items [24], Spanish version [84], e.g., ‘People in our company actively attack problems’; α = 0.92. Respondents answered using 5-point Likert scales ranging from 1 (completely disagree) to 5 (completely agree).

Team work engagement was assessed by nine items [17], e.g., ‘When working, my team feels full of energy’; α = 0.92. Respondents answered using 7-point Likert scales ranging from 0 (never) to 6 (always).

Team Initiative was assessed by seven items [23], e.g., ‘People in our team usually do more than they are asked to do’; α = 0.89. Respondents answered using a 6-point Likert scales ranging from 1 (completely disagree) to 5 (completely agree).

Because team initiative and team work engagement were defined at the group level, and climate for initiative was defined at the organizational level, we assessed within-team agreement (and within-organizations agreement for climate for initiative) by means of the Average Deviation index (AD henceforth) proposed by Burke, Finkelstein, and Dusig [85]. As Burke and Dunlap [86] recommend, to interpret AD values, we used the criterion of AD < c/6, where c is the number of response alternatives. Climate for initiative was assessed with a 5-point Likert-type scale, and so we concluded that there was within-organization agreement when the AD values were ≤0.83. Team initiative was assessed with a 6- point Likert scale and so we concluded that there was within-team agreement when AD values were ≤1. Team work engagement was assessed with a 7-point Likert scale and so we concluded that there was within-team agreement when the AD values were ≤1.16. The mean climate for initiative AD value was 0.45 (SD = 0.19); the mean team work engagement AD value was 0.42 (SD = 0.17) and the mean team initiative AD value was 0.59 (SD = 0.28)

Finally, we carried out a one-way analysis of variance (ANOVA) to ascertain whether there was statistically significant between-team difference in team initiative and team work engagement and significant between-organization difference in climate for initiative. This analysis revealed a significant between-organization difference in climate for initiative, *F*(50, 198) = 5.15, *p* < 0.000. There was also a significant between-team difference in team initiative, *F*(75, 173) = 2.28, *p* < 0.000, and in team work engagement *F*(75, 173) = 2.24, *p* < 0.001. 

#### 2.2.2. Measures from Leaders

Productivity was assessed by four items [87], e.g., ‘My team is very efficient in getting maximum output from the resources (money, people, equipment, etc.) we have available’; α = 0.72. Respondents answered using 6-point Likert scales ranging from 1 (completely disagree) to 5 (completely agree).

Innovation was assessed by one radical innovation item [64]: ‘Radical innovation gives rise to fundamental changes in the activities of an organization or industry with respect to current practices. It poses new questions, develops new technical and commercial skills, and new ways of resolving problems.” How would you describe the level of radical innovation in your organization? “(a) No major innovation at all” (score of 0). “(b) same or very similar innovation adopted by competitors” (score of 1). “(c) similar innovation to the ones adopted by other firms in the same industry but the firm’s innovation differs in identifiable ways from innovations of other firms.” (score of 2). “(d) similar innovation to the ones adopted in other industries” (score of 3). “(e) innovation fundamentally new to the firm” (score of 4) “(f) innovation fundamentally new to the market” (score of 5)’.

### 2.3. Data Analyses

First, we performed a Confirmatory Factor Analysis (CFA) using AMOS 23 to test between three models: (1) a one-factor model where all the constructs are the expression of a single latent factor; (2) a seven-factor model where all the factors (group identity, organizational identity, climates for initiative, work engagement, team initiative, productivity and innovation) are independent; and (3) a seven-factor model where all the factors are correlated. If the seven-factor model provides a better fit than the model with one factor, this provides evidence that common method variance is less prevalent. Second, we computed mediation models to test H1a, H1b, H1c and H2a, H2b, and H2c. The direct and indirect effect involved in this mediator model were tested using the bias- corrected (BC) bootstrap confidence interval method as implemented in PROCESS from SPSS [88]. We use linear regression to assess H3.

Finally, maximum likelihood estimation methods of structural equation modeling (SEM) as implemented by AMOS [89] were used to test the three competing models presented in Figure 1. These were then compared in terms of various goodness-of-fit indices (absolute and relative indices and parsimony indices). The absolute goodness-of-fit indices were (1) the χ2 goodness-of-fit statistic and (2) the root mean square error of approximation (RMSEA). The computation of relative goodness-of-fit indices is highly recommended because the χ2-test is sensitive to sample size [90]. Accordingly, the relative goodness-of-fit indices were calculated with the comparative fit index (CFI) [91]. A parsimony and comparative index were also calculated with Akaike Information Criterion (AIC) [92]. For the RMSEA, values smaller than 0.08 are considered to indicate an acceptable model fit [93]. For the relative fit index (CFI), values greater than 0.90 are considered to indicate a good fit [94]. For the AIC index, which is an index to compare non-nested competing models, the lower the index is, the better the fit of the model and we tested four mediation models that comprised all the study hypotheses. The direct and indirect effect involved in this proposed mediator model with two mediators operating in serial were tested using the bias- corrected (BC) bootstrap confidence interval method as implemented in PROCESS from SPSS [88].

## 3. Results

### 3.1. Data Analyses

Table 2 presents the means, standard deviations, intercorrelations and Cronbach’s αs for all the study variables. Table 3 shows the regression analyzes for productivity and innovation.

We compared the three confirmatory factor analysis models. The fit of the model with seven correlated factors (group identity, organizational identity, climates for initiative, work engagement, team initiative, productivity and innovation) (χ^2^ = 2208.459; df = 799; RMSEA = 0.084; CFI = 0.843; AIC = 2500.459) was better than that of the model with four independents factors (χ^2^ = 2687.293; df = 820; RMSEA = 0.098; CFI = 0.783; AIC = 2937.29) and also better than the fit of the one-factor model (χ^2^ = 5701.554; df = 819; RMSEA = 0.154; CFI = 0.455; AIC = 5953.554). H1—that team identification will be positively related to team work engagement (H1a), that team work engagement will be positively related to team initiative (H1b) and that the indirect positive relationship of team identification with team initiative mediated by team engagement (H1c)—was tested using PROCESS, a SPSS macro created by Hayes [88]. This macro relies on the resampling method of bootstrapping, a procedure that provides an estimate of the indirect effect in the population by resampling the data-set k times (5000 iterations in this study) in order to obtain the indirect effect’s sampling distribution and confidence intervals (CIs). An estimate is considered statistically significant if its 95% CI does not include zero.

Consistent with H1c, the mediation model showed an indirect effect of team identification on team initiative through team work engagement, B = 0.045, SE = 0.016 95% CI [0.009, 0.082]. In line with H1a there was also a significant direct effect between team identification and team work engagement (B = 0.092, SE = 0.038, *p* = 0.016), and in line with H1b there was a significant direct effect between team work engagement and team initiative (B = 0.487, SE = 0.032, *p* < 0.001). 

The same process was carried out to test H2. Consistent with H2c, there was a significant indirect effect of organizational identification on team initiative through climate for initiative (B = 0.049, SE = 0.019 95% CI [0.021, 0.083]) and there was also a direct significant effect between organizational identification and climate for initiative (as predicted by H2a; B = 0.118, SE = 0.040, *p*= 0.003), and a direct significant effect between climate for initiative and team initiative (as predicted by H2b; B = 0.420, SE = 0.042, *p* = 0.000).

### 3.2. Modeling Testing

**Figure 2:**
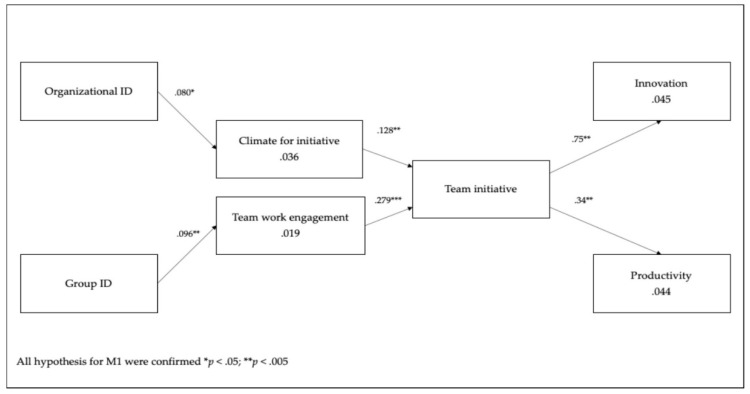
Path Model.

Model 1 (M1) showed an acceptable fit to the data, with all the fit indices meeting their respective criteria (see Table 4). This research model was compared to the two alternative models. The fit indices of models M2, M3 were less good than those for M1. All the paths of Model 1 were significant (*t* > 1.96) (c.f. Figure 2).

Support for Model 1 indicated that team initiative at the group level was related to productivity and innovation. At the same time, organizational identification was positively related to climate for initiative and that in turn was positively related to team initiative; while group identification was positively related to team work engagement that in turn was positively related to team initiative. Model 1 explained 37.9% of the variance of team initiative and 4.4% of the variance of productivity and 4.5% of the variance of innovation. 

The hypothesized mediating role of team initiative and organizational (climate for initiative) or group (team work engagement) antecedents between identity (organizational or group) and performance (productivity and innovation), was also tested using PROCESS. The first mediation model, at the organizational level and with productivity as outcome, identified an indirect effect of organizational identification on productivity through climate for initiative and team initiative (B = 0.012, SE = 0.006 90% CI [0.005, 0.024]). The second mediation model, like the first mediation model but with radical innovation as the outcome, identified an indirect effect of organizational identity on innovation through climate for initiative and team initiative (B = 0.022, SE = 0.014 90% CI [0.006, 0.053]). The third and fourth models, at the group level, identified an indirect effect of group identity on productivity (third model: B = 0.009, SE = 0.056 90% CI [0.002, 0.024]) and innovation (fourth model: B = 0.026, SE = 0.016 90% CI [0.004, 0.071]) through team work engagement and team initiative.

## 4. Discussion

The aim of the present research was to examine the relationship between organizational and team-based identification and organizational innovation and productivity. We also sought to explore the mediating role of climate for initiative, team work engagement and team initiative. 

Consistent with H1 and H2, our findings pointed to the role of social identity processes in explaining the process through which individuals come to show initiative on behalf of the groups and organizations to which they belong [14]. On the one hand, team identification predicted team engagement responses and team initiative, confirming results previously obtained at the individual level [48]. This is consistent with the proposition that social identification motivates people to engage in organizational behaviors on behalf of their teams with a view to advancing those team’s interests [46,47,49]. At the same time, organizational identification predicted team initiative through the climate for initiative. As noted in the Introduction, climate for initiative is an organizational-level variable related to the design of formal and informal practices and procedures that are support proactivity and persistence towards work [21]. In this sense, climate for initiative can be seen to originate in high social identification which leads people to internalize norms and attributes associated with proactive group behaviors (in ways suggested by Blader & Tyler [46]. Our findings also support H3 in so far as team initiative predicted both innovation and productivity. 

Support for the hypothesized model speaks to the suggestion that social identity contributes to team efficiency by providing a psychological platform for team initiative. More generally, we can see that social identification—at both the organizational and team level—predicts the team initiative that drives innovation and productivity in organizations.

### 4.1. Implications and Practical Applications

At a theoretical level, our study provides a model of antecedents and consequents of the personal initiative in workteams, which also makes a case for the value of studying collective phenomena at both an organizational level and a team level. Moreover, this multilevel model integrates two concepts which have previously been studied separately—work engagement and personal initiative—and shows how these can both be seen to derive from (different forms of) social identification. Going forward, this model may be useful for researchers looking to investigate team efficiency using collective constructs. At a practical level, the results also suggest that organizations which want to improve their outcomes should promote and support organizational structures which promote and build social identification at both organizational and team levels. This might be achieved through acts of identity entrepreneurship that encourage and support team work engagement, and which establish clear objectives for the team and which team members themselves are involved in elaborating.

### 4.2. Limitations and Future Research

As with all research, our study had several limitations. One of these resulted from our use of a convenience sample, in which there was a high representation of organizations linked to the knowledge economy. As a result, it remains to be seen whether the observed patterns would generalize to other organizations in other sectors. Second, our use of a cross-sectional design means that it is not possible to infer causal relationships between the variables. In future research it would clearly be preferable to gather longitudinal data. Finally, our use of subjective measures of performance may have introduced measurement error although we took procedural and statistical steps to minimize them. Accordingly, in future studies it will be important to use objective measures where possible to assess innovation and productivity. In addition, there may be value in developing a multi-item measure to assess radical innovation.

## 5. Conclusions

This research applied the Social Identity Approach to organizational psychology to the analysis of initiative and work engagement in workteams. More specifically, we put forward a model which hypothesized relationships between organizational and team identification and productivity and radical innovation. This model also proposed that these relationships are mediated by the climate for initiative, team work engagement, and initiative at the team level. 

Given that they were conceptualized as collective phenomena, the data referring to the predictive variables in this study were analyzed using the average deviation index in order to check the degree of consensus in each workteam before using the aggregated data. Furthermore, an Analysis of Variance (ANOVA) was conducted to establish whether the aggregated data were significantly discriminating between teams and between organizations. Following this, a Confirmatory Factor Analysis (CFA) was carried out in which the proposed 7-factor model (organizational identification, group identification, team work engagement, climate for the initiative, team initiative, productivity and radical innovation) had the best fit with the data. Subsequently, analysis supported both H1 in showing that identification with the team predicted team initiative as mediated by team work engagement, and H2 in showing that identification with the organization predicted climate-mediated team initiative for initiative. H3—that team initiative is positively related to productivity and radical innovation—was also supported by regression analysis. Finally, the model as a whole was tested using structural equation modelling. This had an acceptable fit with the data and was superior to two key alternative models.

In line with previous research [23], our results support the claim that initiative can be conceptualized not only as an individual characteristic or as an aspect of organizational climate [24] but also as an emergent team-level phenomenon that has a bearing on key organizational outcomes such as productivity and radical innovation. In addition, our results indicate that social identification underpins the initiative of workteams via two different pathways. On the one hand, team identification influences team initiative via team work engagement; on the other hand, organizational identification influences team initiative via climate for initiative. Furthermore, team work engagement appears to be a powerful antecedent of team initiative. Together, these patterns point to a novel set of pathways through which social identities in the workplace have a potent impact on key organizational outcomes.

## Figures and Tables

**Table 1:** Sample characteristics.

Variables	M (SD)	N	Percent
Participants (N = 327)			
Gender			
* Men*		169	52%
* Women*		158	48%
Age	41.1 (8.92)		
Education Degree			
* University Degree*		281	86.1%
* Secondary Vocational*		34	10.5%
Organizations (N = 50)			
Organization sectors			
* Consultancy*		21	42%
* Education Services*		8	16%
* Health Services*		5	10%
* Local Development*		5	10%
* Industry*		4	8%
* Insurance*		3	6%
* Logistic*		2	4%
* Software*		1	2%
* Maintenance Services*		1	2%
Organization Size			
* Small Family Businesses*		20	38.7%
* Multinational Companies*		19	37.8%
* Medium Size Companies*		11	22.3%
Work Teams (N = 76)			
* Team Size*	5.83 (5.31)		
* Team Member*		251	76.8%
* Team Leader*		76	23.2%

**Table 2:** Descriptive statistics and correlations.

Variables	M	DT	1	2	3	4	5	6	7
1. Organizational Id.	3.43	1.21	(0.942)	0.869 **	0.184 **	0.152 *	0.038	−0.018	0.051
2. Group Id.	3.44	1.42		(0.962)	0.121	0.153 *	0.090	0.048	0.072
3.Climate for initiative	3.65	0.774			(0.924)	0.490 **	0.534 **	0.049	0.199 **
4. TWEngagement	4.35	0.854				(0.925)	0.696 **	0.159 *	0.174 **
5. TInitiative	4.12	0.596					(0.892)	0.229 **	0.231 **
6. Productivity ^a^	4.21	0.526						(0.726)	0.276 **
7. Radical innovation ^a^	4.10	1.57							

N = 251. Cronbach’s α in the diagonal, between brackets. ^a^. Leader measure, ** *p* < 0.01; * *p* < 0.05.

**Table 3:** Regression analysis.

Variables	B	SE	t	*p*-Value
Productivity	0.230	0.071	3.66	0.000
Radical innovation	0.231	0.164	3.70	0.000

**Table 4:** Model fit.

	χ^2^	*df*	RMSEA	CFI	AIC	Δχ^2^	*df*
M1	1698.54	805	0.069	0.896	1978.5		
M2	2266.51	805	0.088	0.830	2548.5	M2-M1 = 567.97 ***	0
M3	2353.78	813	0.090	0.821	2617.8	M3-M1 = 655.24 ***	8

RMSEA = Root Mean Square Error of Approximation; CFI = Comparative Fit Index. AIC = Akaike Information Criterion. *** *p*-value < 0.001.
